# Training and Validating a Machine Learning Model for the Sensor-Based Monitoring of Lying Behavior in Dairy Cows on Pasture and in the Barn

**DOI:** 10.3390/ani11092660

**Published:** 2021-09-10

**Authors:** Lara Schmeling, Golnaz Elmamooz, Phan Thai Hoang, Anastasiia Kozar, Daniela Nicklas, Michael Sünkel, Stefan Thurner, Elke Rauch

**Affiliations:** 1 Bavarian State Research Center, Institute for Agricultural Engineering and Animal Husbandry, Vöttingerstr. 38, 85354 Freising, Germany; stefan.thurner@lfl.bayern.de; 2 Animal Welfare, Ethology, Animal Hygiene and Animal Husbandry, Department of Veterinary Sciences, Faculty of Veterinary Medicine, Ludwig-Maximilians-University Munich, Veterinärstr. 13/R, 80539 Munich, Germany; e.rauch@tierhyg.vetmed.uni-muenchen.de; 3 Mobile Systems, Faculty of Information Systems and Applied Computer Sciences, University of Bamberg, An der Weberei 5, 96047 Bamberg, Germany; golnaz.elmamooz@uni-bamberg.de (G.E.); thai.hoang@uni-bamberg.de (P.T.H.); anastasiia.kozar@tu-berlin.de (A.K.); daniela.nicklas@uni-bamberg.de (D.N.); michael.suenkel@uni-bamberg.de (M.S.)

**Keywords:** behavior recognition, classification, precision livestock farming, accelerometer, gyroscope, grazing

## Abstract

**Simple Summary:**

There are various systems available for health monitoring and heat detection in dairy cows. By continuously monitoring different behavioral patterns (e.g., lying, ruminating, and feeding), these systems detect behavioral changes linked to health disorders and estrous. Most of the systems were developed for cows kept indoors, and only a few systems are available for pasture-based farms. The systems developed for the barn failed to detect the targeted behavior and thereby its changes on the pasture and vice versa. Therefore, our goal was to train and validate a machine learning model for the automated prediction of lying behavior in dairy cows kept on pastures, as well as indoors. Data collection was conducted on three dairy farms where cows were equipped with the collar-based prototype of the monitoring system and recorded with cameras in parallel. The derived dataset was used to develop the machine learning model. The model performed well in predicting lying behavior in dairy cows both on the pasture and in the barn. Therefore, the building of the model presents a successful first step towards the development of a monitoring system for dairy cows kept on pasture and in the barn.

**Abstract:**

Monitoring systems assist farmers in monitoring the health of dairy cows by predicting behavioral patterns (e.g., lying) and their changes with machine learning models. However, the available systems were developed either for indoors or for pasture and fail to predict the behavior in other locations. Therefore, the goal of our study was to train and evaluate a model for the prediction of lying on a pasture and in the barn. On three farms, 7–11 dairy cows each were equipped with the prototype of the monitoring system containing an accelerometer, a magnetometer and a gyroscope. Video observations on the pasture and in the barn provided ground truth data. We used 34.5 h of datasets from pasture for training and 480.5 h from both locations for evaluating. In comparison, random forest, an orientation-independent feature set with 5 s windows without overlap, achieved the highest accuracy. Sensitivity, specificity and accuracy were 95.6%, 80.5% and 87.4%, respectively. Accuracy on the pasture (93.2%) exceeded accuracy in the barn (81.4%). Ruminating while standing was the most confused with lying. Out of individual lying bouts, 95.6 and 93.4% were identified on the pasture and in the barn, respectively. Adding a model for standing up events and lying down events could improve the prediction of lying in the barn.

## 1. Introduction

Precision Livestock Farming (PLF) has gained importance in the dairy sector all over Europe over the last decade. The application of smart farming solutions offers great potential for improving productivity and management on dairy farms. Different studies investigated the automizing activity recognition of humans [1,2,3] or animals [4,5] in real environments. These studies are based on technologies such as smart environment, Internet of Things (IoT), machine learning and big data. The standard strategy applied in these studies is to use sensors and to classify the sensor data into desired activities or behavioral patterns by applying suitable machine learning models on the sensor data. This process is also used in monitoring systems for dairy cows in order to predict the behavior of animals continuously and individually. Triaxial accelerometers, e.g., combined with a magnetometer or a gyroscope, are the sensors widely used for this purpose. As the lifespan of the sensor’s battery is crucial for the successful application of monitoring systems, adjusting the sampling rate can be beneficial [6,7]. As presented by Kamminga et al. [8] and Krause et al. [9], a combination of accelerometer, magnetometer and gyroscope data can provide an orientation-independent dataset that ensures sufficient accuracy of the model, even when a low sampling rate is applied.

Monitoring the health and welfare of dairy cows is time consuming. Increasing herd sizes reduces the amount of time available for the individual animal [10,11]. On a pasture, the control of animals is impeded by distances and environmental conditions. Monitoring systems assist the farmer in supervising the health and welfare of the animals and, thus, reduce the physical workload and increase schedule flexibility [12,13,14]. Based on behavior data, changes can be registered, and the corresponding alerts for the farmer can be generated through management software. Changes in behavior occur, e.g., during estrous and under extreme environmental conditions and with emerging health issues. Behavioral changes caused by diseases often arise before clinical symptoms appear, which allows earlier detection by using monitoring systems compared to visual examination of the animal [15,16,17]. The early identification of commencing health disorders enables timely treatment, resulting in minimized veterinary costs [18], reduced use of antibiotics [19,20] and rapid restoration of the welfare of the animals by limiting the pain and discomfort linked to diseases [21,22,23]. Identifying behavioral changes linked to extreme weather conditions enables the farmer to take preventive measurements in order to reduce the impact on the health and welfare of the animals. A reliable detection of estrous with monitoring systems results in an increased number of successful inseminations, reducing the expenses associated with missed heat events [24,25].

In addition to other behavioral patterns, lying behavior is one of the most significant indicators for estrous and health challenges including emerging health disorders and extreme environmental conditions. Total lying time, duration of lying bouts and the frequency of lying events can be either reduced or increased. On the day of estrous, e.g., dairy cows exhibit an increased level of activity at the expense of lying time [26]. Mayo et al. [27] also found a decrease in the number of lying bouts on the day of estrous. As heat dissipation is increased while standing compared to lying, animals reduce their lying time in favor of standing when temperatures are high [28,29]. Allen et al. [28] also found a decreased duration of lying bouts. Moreover, cows exposed to wet and cold weather conditions or suffering from mastitis lie down less [30,31]. On the other hand, Beer et al. [32] and Weigele et al. [33] found that lame cows show increased lying time and lying bout duration compared to sound cows. In the study of King et al. [16], dairy cows with displaced abomasum, metritis or pneumonia lie down more on the day before diagnosis, accompanied by an increased duration of lying bouts.

Apart from lying behavior, other behavioral patterns are affected by estrous, health disorders and extreme environmental conditions. The changes of those behaviors can serve as indicators used by monitoring systems as well. Reith and Hoy [34] found a relationship between decreased rumination time and estrous. Moreover, high temperatures affect rumination time, making it a useful indicator for heat load in dairy cows [35]. Barker et al. [36] used feeding time as an indicator for lameness. Reliable detections of estrous and early detection of health disorders and challenges are possible by assessing behavioral patterns and their changes individually, but combining multiple behaviors improves the information value and the detection rate. In the studies by Stangaferro et al. [21,22,23], various health disorders were detected before clinical diagnosis based on changes both in rumination time and activity.

In order to detect the behavioral patterns, e.g., lying behavior, monitoring systems can be applied to various parts of the cows’ body. In the study of Borchers et al. [37], different pedometers predicted lying behavior with reasonable accuracy. Bikker et al. [38] showed that accelerometers integrated into an ear tag predicted lying in dairy cows reliably. Benaissa et al. [39] compared the performance of a leg-based and a neck-based system. Both systems performed well in predicting lying behavior, but considering other behavioral patterns, the accuracy of the neck-mounted system was higher.

Offering pastures to dairy cows is linked to various benefits. In addition to improving health [40], the animals’ actions conform more to their natural behavior, expressed by an increased synchrony in feeding and lying behavior in grazing situations compared to the barn [41]. From the consumer perspective, farms with access to pastures reach higher levels of animal welfare [42]. Despite the benefits, in Europe as well as the US, the share of cows with access to pasture declined over the last years [43,44]. In addition to other region and farm specific factors, insufficient grassland constrains offering pasture to dairy cows [44]. Lack of a sufficient amount of grassland combined with rainy winters prevents year-round grazing in Central Europe [43,44]. Therefore, when offering pastures to dairy cows, a combined husbandry system of barn and pastures is practiced.

Most of the systems for monitoring dairy cows that are available on the global market have not been (properly) validated, and when the same sensor is validated in different husbandry systems, performance varies [45]. The models included in the systems for the automated prediction of different behavioral patterns, and their changes perform weakly when applied in the location they were not trained in [46,47]. The fact that offering pastures to dairy cows improves their welfare and that the demand for improved welfare increases while concurrently grazing in some parts of the world is only possible by combining the pasture and barn offers increasing market potential for hybrid monitoring solutions applicable both on pastures and in the barn [44,48].

Therefore, the goal of the presented study was to train and to evaluate a model for the automated prediction of lying behavior in dairy cows kept on pasture as well as indoors. Behavioral data derived from video observations served as ground truth. To build a reliable model, a promising combination of classifier, selected features and data segmentation, i.e., window size and stride, had to be found. The sample frequency was altered in order to reduce energy demand while maintaining high prediction accuracy. The training and validation of a model for the prediction of lying behavior are the first steps towards a system for dairy cows for automated monitoring of behavioral patterns, their changes and, thereby, the early prediction of conditions (e.g., diseases, heat load and estrous) causing those changes.

## 2. Materials and Methods

### 2.1. Data Collection and Labeling

Data were collected on three dairy farms in Upper Bavaria, Germany. All procedures performed followed the EU directive 2010/63/EU and the German Animal Welfare Act. The conducted procedures did not interfere with or deviate from regular farm practices. On all farms, the cows had access to the pasture in the summer (April to October), calving took place seasonally (November to March) and the pastures were managed continuously with the same area being available to the cows at all times.

#### 2.1.1. Farm Management and Animals

On farm 1, the dairy herd consisted of 40 dairy cows that were exclusively Simmental. The mean milk yield in the preceding year was 7397 kg. Data collection was conducted in two rounds of two consecutive days, each in September and October 2018. During the trial, cows were kept on a pasture (17 ha; see Table 1) and milked twice daily at approximately 06.00 and 17.00 h in a herringbone milking parlor. For two hours around each milking time, the cows were given access to a freestall barn with deep straw-bedded cubicles (n = 40). During morning milking, a negligible portion of grass or maize silage mixed with concentrate was fed in the barn. Water was supplied ad libitum via seven troughs on pasture and two in the barn. Several mineral lickstones were available on the pasture and in the barn. Five (round 1) and eight (round 2) lactating cows were randomly chosen from the herd. Only animals in the second to sixth lactation were selected. Cows were clinically healthy and free from lameness, changes in milk composition or any other clinical signs of health disorders. The average parity of the selected animals was 3.4 ± 0.5 (mean ± standard deviation; round 1) and 3.5 ± 1.2 (round 2). The cows were 227 ± 28 and 285 ± 40 days in milk (DIM) on the first day of each round, respectively. The average Body Condition Score (BCS) was 3.2 ± 0.5 and 3.1 ± 0.4 on the first day of each round, respectively. BCS was defined based on the figure by Edmonson et al. [49] modified after Metzner et al. [50].

On farm 2, the dairy herd consisted of 34 dairy cows that were exclusively Simmental. The mean milk yield in the preceding year was 7437 kg. Data were collected on three consecutive days in July 2019. During the day, cows were kept on a pasture (12 ha; see Table 1) with permanent access to a freestall barn with deep litter cubicles (n = 34). Between afternoon and morning milking, which was performed at 16.00 and 07.30 h, the cows were kept on a smaller pasture (3 ha; see Table 1) without access to the barn. Before each milking, a negligible portion of maize silage mixed with concentrate and minerals was fed. Water was supplied ad libitum via five troughs on the day pasture, one on the night pasture and two in the barn. Eleven lactating cows were randomly selected from the herd based on the same criteria applied on farm 1. The average parity of the selected cows was 4.0 ± 0.9, and they were 273 ± 16 DIM on the first day of the trial. Average BCS was 3.0 ± 0.6 on the first day of the trial.

On farm 3, the dairy herd consisted of 52 dairy cows that were mainly Simmental (46 Simmental, 3 Red HolsteinXSimmental, 1 Red Holstein, 1 German Black Pied and 1 German Red Pied). The mean milk yield in the preceding year was 8232 kg. Data were collected on four consecutive days in March 2019. Due to winter weather, the cows were kept in a freestall barn with high cubicles (n = 48) equipped with rubber mattresses. The cows were milked twice a day in a tandem milking parlor at approximately 06.00 and 18.00 h. The cows were fed a mixed ration (containing maize and grass silage, concentrate and minerals) twice a day at approximately 10.00 h and during afternoon milking. Feed remains were removed before the next feeding. Additional concentrate was offered in a computerized feeder to cows with a milk yield of ≥35 kg/d within the last five days. Water was supplied ad libitum via two troughs. Several mineral lickstones were available. Eleven lactating cows were randomly chosen from the herd based on the same criteria applied on the other farms. The average parity of the selected cows was 3.8 ± 1.4, and they were 103 ± 40 DIM on the first day of the trial. The average BCS was 3.8 ± 0.4 on the first day of the trial.

#### 2.1.2. Collection of Sensor and Ground Truth Data

**Figure 1:**
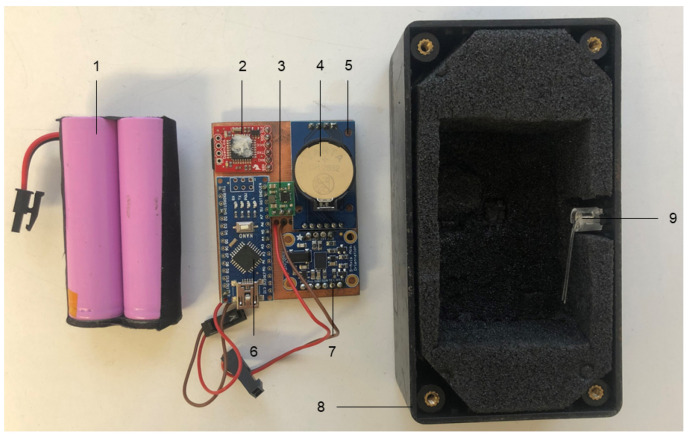
Composition of the prototype of the monitoring system from Blaupunkt Telematics GmbH (Hildesheim, Germany) and the included sensor board: 1—lithium batteries (Samsung ICR18650 26H; Samsung Group, Seoul, South Korea); 2—data logger (SparFun OpenLog ATmega328; SparFun Electronics, Niwot, CO, USA) containing a Secure Digital Memory Card (32 GB; SanDisk; Western Digital Deutschland GmbH, Aschheim, Germany); 3—voltage regulator (S18V20ALV; Pololu Robotics & Electronics, Las Vegas, NV, USA); 4—lithium button cell (CR2032; Varta Consumer Batteries GmbH & Co. KGaA, Ellwangen, Germany); 5—real time clock (DS3231; Maxim Integrated Products, Inc., San Jose, CA, USA); 6—controller (Arduino Nano V3 with CH340; AZ-Delivery Vertriebs GmbH, Deggendorf, Germany); 7—breakout board (9-DOF Absolute Orientation IMU Fusion Breakout; Adafruit, New York, NY, USA) containing the system in the package (BNO055; Bosch Sensortec GmbH, Reutlingen, Germany) combining a 3D accelerometer, a 3D magnetometer and a 3D gyroscope; 8—case of the prototype (Hammond 1591; 133 × 63 × 35 mm; Hammond Manufacturing^TM^, Frankfurt am Main, Germany) with handmade foam; 9—LED to show functionality of the system.

**Figure 2:**
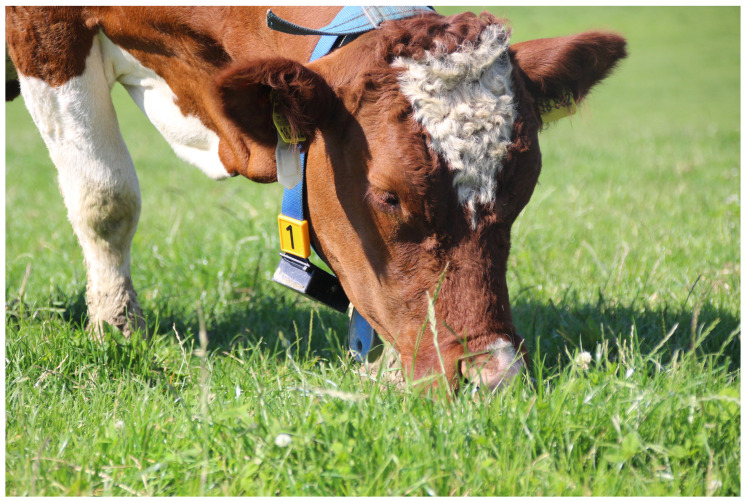
Prototype of the monitoring system (Blaupunkt Telematics GmbH; Hildesheim, Germany) mounted on the collar of a Simmental cow grazing pasture.

On all farms, the selected animals were equipped with the prototype of the monitoring system (see Figure 1; 133 × 63 × 35 mm; 220 g; Blaupunkt Telematics GmbH, Hildesheim, Germany) attached to a collar. The case was located at the lower neck of the animals (see Figure 2). The system contained a sensor from Bosch (BNO055; Bosch Sensortec GmbH, Reutlingen, Germany) including a three-dimensional (3D) accelerometer, a 3D magnetometer and a 3D gyroscope. The settings were selected to measure the linear acceleration (m/s^2^) with the accelerometer and the Euler angle (°) by fusing the values from all nine axes (=NDOF operating mode). Data collection frequency was set to 10 Hz. Raw data were stored on an integrated SD memory card (32 GB; SanDisk; Western Digital Deutschland GmbH, Aschheim, Germany) and downloaded after each round. Two rechargeable lithium batteries (Samsung ICR18650 26H; Samsung Group, Seoul, South Korea) served as a power supply. A General Positioning System (GPS) sensor (NEO-6; u-blox Holding AG, Thalwil, Schweiz) (farm 1) and a real-time clock (RTC; DS3231; Maxim Integrated Products, Inc., San Jose, CA, USA) (farm 2 and 3) were used for time synchronization of the sensor system.

In order to collect ground truth data, the behavior of the animals was recorded with cameras (GoPro HERO5; GoPro, Inc., San Mateo, CA, USA). Superview, 1920 × 1080 pixel and 60 frames per second were set for the recordings. In order to allow continuous recording of the selected animals on pasture, four to five cameras were attached to tripods and repositioned frequently by two to four observers. In order to avoid behavioral disturbance, observers remained at adequate distances from the herd. In the barn, seven cameras were installed at fixed positions. The time of the videos was synchronized with radio-controlled clocks (Hama GmbH & Co KG, Monheim, Germany) that were visible in the camera image. Since the animals on all three farms became used to wearing the collar with the prototype very quickly, the observation begun around one hour after attachment.

Following the observations, the videos were labeled, i.e., the behavior of the observed cows was defined at all times based on an ethogram (see Table 2) by one trained observer. The ethogram consisted of exclusive behavioral patterns such as lying down, standing up, standing, walking, grazing and activities that overlapped with behaviors such as feeding, chewing, ruminating, drinking and others. Behavior definitions were derived from Martiskainen et al. [51], Reiter et al. [52] and Werner et al. [53]. In order to identify the animals in the video, numbers were sprayed on the flank, and the individual coat patterns were used. Data from all animals on all days from farm 1, all animals on one day from farm 3 and three animals on one day from farm 2 were labeled in detail. For the remaining data, differentiation was only made for lying and non-lying, with non-lying including all behaviors except for lying. Labeled video data were regarded as ground truth. Labeling was performed by one observer exclusively. In order to assess observer reliability, 20% (=26 h) of video data from farm 1 were labeled twice by the same observer. Time frames of ten minutes were randomly distributed among rounds, days, animals and time of day. The outputs of both labeling processes were compared second by second.

### 2.2. General Process of Data Acquisition and Model Development

**Figure 3:**
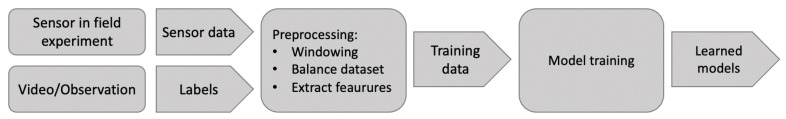
Procedure of the training phase that consists of two steps: preprocessing and model training. The goal of this step is to create prediction models that can be applied to new sensor data.

The main goal of the developed prediction framework is to build behavior recognition models in order to analyze the dairy cows’ behavior. This framework includes two phases: training and validation. In this paper, we focus on lying behavior, but the framework can generally recognize a bigger variety of the dairy cows’ behavior. The goal of the training phase (Figure 3) is to create supervised prediction models that can be applied to new sensor data, which are unknown to the model. In this phase, the sensor data from a field experiment are labeled by a domain expert who observed the videos that were made during the experiment.

The training phase consists of two main steps: preprocessing and model training. Since the data coming from sensors contain noisy data or inconsistent timestamps, they require the application of preprocessing techniques. These techniques, e.g., noise reduction and time synchronization, etc., are needed to create necessary datasets for training and to enhance the accuracy of the learned models. During the training of the model, the data are classified by a learning algorithm where the classes are the different behavioral patterns of dairy cows (=labels). The output of the training phase consists of learned models, which were created after the model training. These models comprise training dataset specifications, e.g., training time and number of cows, which include the following: which preprocessing techniques were applied, what should be predicted (e.g., lying vs. non-lying), which learning algorithm is executed (e.g., random forest with defined parameters), which dataset is used for testing, which evaluation metrics are used (e.g., accuracy and recall, etc.) and the result achieved (e.g., 90% accuracy). The accuracy was measured as a ratio between all correct outcomes and all possible outcomes using the Python 3 package scikit-learn 0.22.2 (https://scikit-learn.org/0.22/, accessed on 2 September 2021).

**Figure 4:**
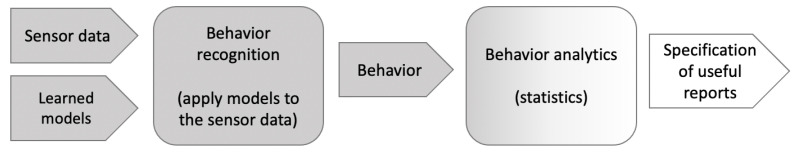
Procedure of the prediction phase, which includes applying new data to the trained models for validation of their prediction performance.

During the prediction phase (Figure 4), new sensor data are applied to the learned models in order to obtain useful reports for the end user. The goal of this phase is to check the validity of the trained models which were created in the previous phase. Therefore, new unlabeled data will be applied to the learned models in order to create insights. These insights will be cross-checked by a domain expert in order to determine the validity of the trained model’s outcome. Valid reports will be classified as useful and sent to the end user. In case no useful reports can be developed, the model will be redirected to the first phase, where either new or improved models will be created. In this study, we evaluated whether the behavior recognition is suitable for higher level behavior analytics. Thus, in Figure 4, the steps “behavior analytics” and “specification of useful reports”, which are marked white, were performed only partially (only the learned model was evaluated but not the reports) or were disregarded (no reports were created because there was no end user).

### 2.3. Feature Selection and Model Development

The selection of a model or the combination of a feature set with a corresponding classifier is perhaps the most important phase in building a behavior recognition system, as it was also shown by Bersch et al. [54].

#### 2.3.1. Feature Selection

For behavior recognition problems, which rely on time-series sensor data, data segmentation is the most common approach for partitioning a data stream into time-based windows. Those data segments are used for feature extraction and selection. In our study, instead of classifying every data point from the sensor data stream, a segmentation technique was used to divide the data into windows of data points from which the features were computed. The selection of the right segmentation technique, in terms of window size and stride, is crucial to the systems’ performance. At the level of a time-based window, the length of window indicates the volume, while the frequency of a window update (stride length) reflects the velocity in collecting the sensor data. In order to find the best segmentation strategy, we assessed window sizes from 1 to 20 s, with strides from 25 to 100%. A feature was derived by applying a mathematical function on a series of values of a sensor axis (or derived axis) over a window of data (Equation (1)).

(1) $$\underset{Window}{\underset{︸}{\left\{x_{1},x_{2},\ldots ,x_{n}\right\}}}\overset{f}{\to }Feature$$

Generally, features in the field of behavior recognition can be grouped into three major types (time-domain, frequency-domain and discrete-domain), which were presented in Figo et al. [55]. Out of the three types, time-domain and frequency-domain are most commonly used in research concerning behavior recognition [2,3,7].

The problem of sensor orientation has been recognized in many studies which showed that the variability in sensor orientation attached to dairy cows brings about significant errors in classifier performance. A notable research study, which also tackled this problem, is the one from Kamminga et al. [8], where a feature set that is robust relative to the sensor orientation was explored and evaluated using various classifiers.

The effect of sensor data with different orientation on the various classifiers’ performance was verified by implementing two feature sets. The first set was known to be sensitive to sensor orientation and comprised 24 features, which were formed by applying four functions, i.e., max, mean, median and standard deviation over the six axes of the two sensors. The second set was insensitive to sensor orientation and was constructed by applying 18 functions, namely min, max, mean, median, standard deviation, interquartile range, root mean square, mean crossing rate, kurtosis, skewness, spectral energy, peak frequency, frequency domain entropy and the first five feature frequency profile components on two derived axes: the magnitudes of the 3D accelerometer and the NDOF vector, respectively. Features were selected based on their importance by using the Python 3 scikit-learn 0.22.2 package (https://scikit-learn.org/0.22/, accessed on 2 September 2021).

#### 2.3.2. Model Selection and Development

During the model selection process, random forest, decision tree, support vector machine and naive Bayes were selected (Table 3) for the experiment based on their previous success in the prediction of cow behavior, including lying [39,51,56,57]. The features used were consistent between the models.

Table 3 shows the classifiers together with the corresponding selected hyperparameters. The hyperparameters were tuned by exhaustive grid search method in order to find the optimized parameters for each model. A 10-fold cross-validation was used to estimate the performance of the model in the train-validation phase. The framework implementation and model parameter comparison were implemented using Python 3 and its scikit-learn 0.22.2 package (https://scikit-learn.org/0.22/, accessed on 2 September 2021).

### 2.4. Postprocessing

Filtering is a crucial part of data postprocessing. It displays a clear sequence of procedures or steps that must be followed in order to obtain reasonable and understandable results. The data that are the result of a knowledge acquisition (prediction) algorithm are usually noisy and sometimes inconsistent. In the case of the mentioned framework, filtering was applied to avoid unnecessary small breaks between similar or same activities. From an ethological point of view, filtering is supported by the fact that behavior that occurs in bouts, such as lying behavior, requires an interbout criterion [58]. An interbout criterion seperates short interruptions of a behavioral pattern between events from interruptions between bouts. After extensive parameter comparison, the decision was made towards 60 s time period. For example, if lying lasted for a considerable amount of time after the prediction part the behavior then stopped for less than 60 s and then started again, it was likely that it was either a prediction mistake or an unnecessary small side behavior (noise) that could have been emitted. By applying the filter, sequences of lying or non-lying, with duration less than 60 s, were filtered and added to the behavior predicted for the time period preceding the sequence. The filter not only helps specifying individual lying bouts but also improves overall prediction accuracy.

### 2.5. Evaluation of the Model and Statistical Analysis

Data which were not used for the training of the model served as a basis for the evaluation. In order to assess performance, the outputs of the model and the ground truth were compared per second. Pure data (i.e., without overlapping) and data points with overlapping behaviors were used for the evaluation of the model. Data points (=seconds) correctly identified as lying behavior were defined as true positive (TP), and data points correctly identified as non-lying behavior as true negative (TN). Data points of lying behavior that were classified as non-lying behavior by the model were considered as false positive (FP), and data points of non-lying behavior that were classified as lying by the model were considered as false negative (FN). Sensitivity, specificity and accuracy, as well as positive and negative predictive values, were calculated according to the following equations.

(2) $$\mathrm{Sensitivity}=\frac{\mathrm{TP}}{\left(\mathrm{TP}\;+\;\mathrm{FN}\right)}$$

(3) $$\mathrm{Specificity}=\frac{\mathrm{TN}}{\left(\mathrm{TN}\;+\;\mathrm{FP}\right)}$$

(4) $$\mathrm{Accuracy}=\frac{\left(\mathrm{TP}+\mathrm{TN}\right)}{\left(\mathrm{TP}\;+\;\mathrm{TN}\;+\;\mathrm{FN}\;+\;\mathrm{FP}\right)}$$

(5) $$\mathrm{Positive}\;\mathrm{predictive}\;\mathrm{value}=\frac{\mathrm{TP}}{\left(\mathrm{TP}\;+\;\mathrm{FP}\right)}$$

(6) $$\mathrm{Negative}\;\mathrm{predictive}\;\mathrm{value}=\frac{\mathrm{TN}}{\left(\mathrm{TN}\;+\;\mathrm{FN}\right)}$$

Performance parameters were calculated in total as well as for every animal on every day separately. Lying bouts were considered as identified when an overlap between ground truth and the model output occurred. Regarding duration, only lying bouts without interruption caused by missing visibility in the video image or a gap in the sensor data were considered. Only bouts completely recorded both in ground truth and model output were compared.

The influence of animal, day, farm and location (pasture vs. barn) on the sensitivity, specificity and accuracy of the model was analyzed with generalized additive models by using the mgcv package in RStudio 1.3 (RStudio, Inc., Boston, MA, USA; [59]):

(7) $$Y_{ijkl}=f_{i}+d_{j}+l_{k}+a_{l}+e_{ijkl}$$

where

*Y*${}_{ijkl}$ = value of performance (sensitivity, specificity and accuracy);
  *f*${}_{i}$ = fixed effect for farm;
  *d*${}_{j}$ = fixed effect for day;
  *l*${}_{k}$ = fixed effect for location (pasture/barn);
  *a*${}_{l}$ = repeated effect for animal;
  *e*${}_{ijkl}$ = random residual.

Farm, day and location were set as fixed effects. The animal was set as a random effect. In order to further analyze the influence of the day within one farm, the same model was used, leaving out farm and location as fixed effects. The difference between the duration of lying bouts in ground truth and model output and the difference between performance during the day compared to the night was evaluated using Wilcoxon test. Kendall’s correlation coefficient was calculated to assess the relation between standing time and accuracy, sensitivity and specificity. The correlation coefficient was categorized as follows: 1 = perfect; 0.9 to 0.7 = strong; 0.6 to 0.4 = moderate; and <0.4 = weak correlation.

In order to evaluate the duration of lying behavior over 24 h and the differences between husbandry system, the model was applied to one day (the day with the most data available) on each farm. Only animals with <10% of missing sensor data according to Elischer et al. [47] were included in the evaluation. For the comparison of lying time between husbandry systems, a one-sided ANOVA [60] and a post hoc test with Bonferroni correction was performed [61]. In order to verify homogeneity of variances, a Levene’s test [62] was performed and residuals’ distribution was tested for normality by Shapiro–Wilk test [63]. With a Wilcoxon test, we compared the lying duration during the day (06.00 to 18.00 h) and the night (18.00 to 06.00 h) for each farm separately. Analysis of differences in lying time between husbandry systems was performed in R.Studio 1.3 (RStudio, Inc., Boston, MA, USA). Where applicable, *p*-values < 0.05 were considered as significant.

## 3. Results

### 3.1. Collected Data

In total, 1864.0 h of sensor data and 542.2 h of ground truth data were collected, resulting in 538.7 h of complete datasets with concurrent sensor and ground truth data. The data from one animal, each on farm 1 and farm 3, were disregarded because the cows showed symptoms of estrous within the observation round. The data from three animals from farm 1 were used for the training of the model (see Table 4). Regarding training data, only pure data, i.e., without other activities (e.g., ruminating and social behavior) overlapping, were used. Disregarding the data from the animals in heat and the three animals from farm 1, which were used for training of the model, 476.2 h of datasets were left for the evaluation of the model. The performance of the model on pasture and in the barn was assessed separately. On pasture, 238.7 h of datasets were collected (farm 1 and farm 3), and 237.5 h of datasets were collected in the barn (farm 2 and farm 3). The amount of collected data differed between farms, rounds and days (see Table 5) due to different availability and runtime of sensor systems. On farm 1 and 2, all data were collected during the day, i.e., in the time period from 06.00 to 18.00 h. On farm 3, 24.6 h of data were collected in the time period between 18.00 and 06.00 h, and the remaining data were collected during the day.

### 3.2. Observer Reliability

Of all data, 20% (equalling 26 h) of data from farm 1 were coded twice. The agreement between the first labeling and the second labeling was 94.8% for detailed labeling and 100% for lying and non-lying.

### 3.3. Model

In order to evaluate the four classifiers (random forest, decision tree, naive Bayes and support vector machine) selected from Section 2.3.2, the sensor data from six dairy cows from two different farms (three from farm 1 and three from farm 2) were used to train and evaluate the models. Data distribution can be observed in Table 4. Since the orientation of the sensors used on the first farm was different from the second farm, the datasets were mixed so that it was possible to create training and testing datasets, which contained different orientation sensor data.

**Figure 5:**
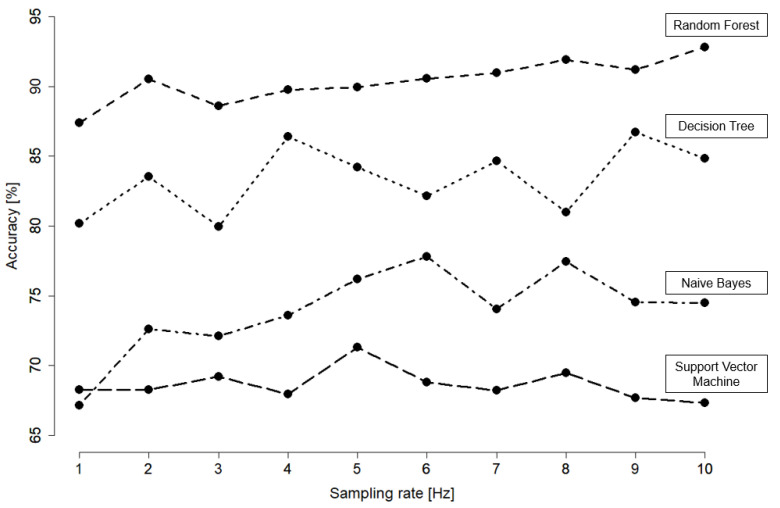
Accuracy comparison of the different classifiers (random forest, decision tree, naive Bayes and support vector machine) based on different sampling rates (1–10 Hz). The postprocessing filter was not applied for this comparison.

**Figure 6:**
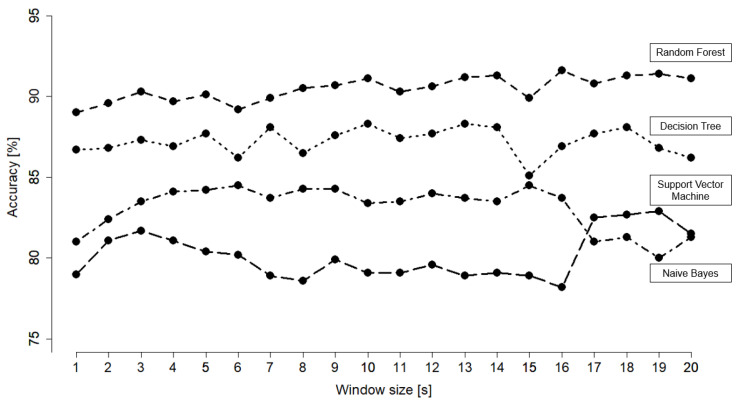
Accuracy comparison of the different classifiers (random forest, decision tree, naive Bayes and support vector machine) based on different window sizes (1–20 s). The postprocessing filter was not applied for this comparison.

The comparison that can be observed in Figure 5 and Figure 6 showed that random forest was the classifier that yielded the highest accuracy in comparison to the other three classifiers. Since the sampling rate has effects on the energy consumption of the sensor system, we studied the effects of reducing the sampling frequency in the data from its original rate of 10 Hz down to 1 Hz. However, from Figure 5 we can observe that 10 Hz proved to be the most effective sampling rate, which brought the highest accuracy for random forest. Hence, we used the original rate in further works.

The comparison of the different window sizes can be found in Figure 6. The window size parameter was chosen to be 5 s as a trade-off between the accuracy and the prediction latency.

In addition to window size, the stride of windows had to be defined. We compared a stride of 25, 50 and 100% (results not shown), and 100% was found to be the most accurate for random forest and a window size of 5 s.

The two sets of features combined with four classifiers presented previously brought eight different models for the experiment. Since it was found to achieve the highest accuracy, the following section will present the result that was obtained using the random forest classifier. The two sets of features selected in Section 2.3.1 combined with random forest created the following two models (see Table 6).

Firstly, we used the data of six dairy cows in order to implement the subject cross-validation to observe how different orientation sensor data affect the model’s performance. The training and testing data were set up for evaluation in the following manner: only the cows that did not participate in training were allowed to participate in the testing process.

The following result was obtained when the two models were both trained and tested on data from farm 1 (=sensor data with the same orientation). From Table 7, it can be concluded that model 1 experienced overfitting and instability, unlike model 2. However, both models produced fairly high accuracy.

Table 8 shows the performance of the two models when they were trained on data from farm 1 but tested on data from farm 2 (different sensor orientation). It is clear that the test accuracy produced by model 1 was experiencing both considerable decrease and instability while, model 2 remained stable in performance. This result once again confirmed the robustness of the model having an orientation-independent 36 feature set, which was derived by applying 18 functions over the magnitudes of the accelerometer and the NDOF vector as described in Section 2.3.1. This 36 feature set was, therefore, selected as the feature set for the following evaluation.

The conducted experiment resulted in two datasets with data from different sensor orientation. This experiment showed slight improvement in the overall accuracy for the selected model. Moreover, it was found that the training data from only three dairy cows were enough for the model to gain sufficient generalization. In further experiments, we found that selecting more than three cows did not improve the accuracy of the model (data not shown). As a result, the most feasible model was obtained by training the 36 feature random forest model with the sensor data from three dairy cows from the same farm (farm 1).

### 3.4. Performance of the Model

Overall sensitivity, specificity and accuracy of the model with applied filter were 95.6%, 80.1% and 87.3%, respectively. Lying behavior was predicted with a positive predictive value of 80.5% and a negative predictive value of 95.5%. Overall accuracy on pasture (93.1%) was higher than the accuracy in the barn (81.4%). Within farm 2, the model predicted lying behavior with an accuracy of 91.0% on pasture and 86.3% in the barn.

Total median accuracy per animal and day was 91.7%. Median accuracy per animal and day on pasture and in the barn was 95.3% and 84.4%, respectively. Median accuracy per animal and day on pasture was significantly higher than in the barn (*p* < 0.01). In total, the farm had a significant effect on the median accuracy per animal per day (*p* < 0.01). Highest accuracy was achieved on farm 1, followed by farm 2. On farm 3, the lowest accuracy was attained. Although there were differences in accuracy between animals, there was no significant effect of the animal on the accuracy per animal and day (*p* = 0.31).

In general, the day had a significant effect on the accuracy per animal and day (*p* < 0.05). The comparison between the performance of the model within one farm showed significant differences between days on farm 1 and 3. On farm 1, the accuracy per animal on day 2 was significantly lower than on day 1 and day 4 (*p* < 0.05). There were no significant differences between the other days. On farm 3, accuracy per animal on day 1 was significantly lower than on day 2 (*p* < 0.05). There were no significant differences between the other days.

On farm 3, a part of the data was collected in the time period between 18.00 and 06.00 h. No difference was found between the accuracy per animal and day during the night and during the day (*p* = 0.46). In general, the model overestimated lying time at the expense of non-lying time. In total, 54.7% of the observed time was classified as lying by the model, while lying time shared 46.1% in ground truth.

Lying behavior falsely classified as non-lying (FN) amounted to 5.5 h on pasture and 4.2 h in the barn, equaling 2.3 and 1.8% of the observed time. Of non-lying behavior, 11.0 (4.5%) and 40.0 h (16.8%) were misclassified as lying (FP) outdoors and indoors, respectively. Of the FP time recorded on pasture and in the barn, 4.0 and 11.1 h of the FP were labeled and evaluated in detail. On pasture, 1.8 h of the FP time was located before, after or in between lying periods. The remaining 2.2 h could not be linked to a lying event. With 58.8%, ruminating while standing was the behavioral pattern confused the most with lying, followed by standing and walking with 20.0 and 8.9%. In the barn, 6.9 h of the FP time was located before, after or in between lying periods. The remaining 4.2 h could not be linked to a lying event. With 53.8%, ruminating while standing was the behavioral pattern confused the most with lying as well. Standing in the cubicle (without ruminating) was the second most common pattern falsely classified as lying with 25.6%.

Since standing (in the cubicle) was the behavior confused the most with lying, specificity as well as accuracy per animal and day was negatively correlated (τ = −0.53 and τ = −0.60; *p* < 0.01) with the share of standing behavior of the observed time. While standing shared 9.9% of the total observed time on pasture, 47.6% standing time occurred in the barn.

**Figure 7:**
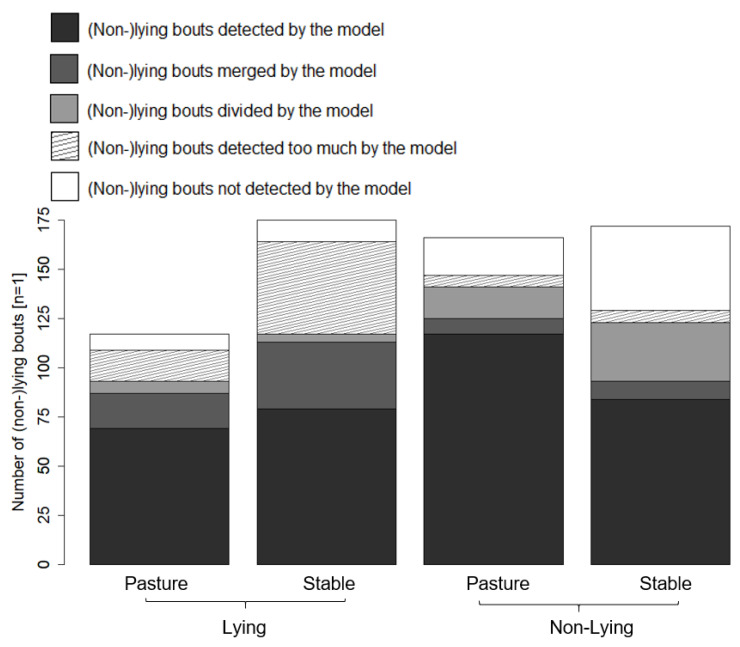
Detection of individual lying bouts and non-lying bouts including detected, merged, divided, missed and added bouts.

The detection of individual lying bouts and non-lying bouts by the model is presented in Figure 7. Of the lying bouts, 93.3% and 96.4% and 88.7% and 75.7% of the non-lying bouts were identified by the model on the pasture and in the barn, respectively. Three of the lying bouts on pasture, one of the lying bouts in the barn and one non-lying bout on pasture lasted < 60 s, i.e., the duration was lower than the filter of 60 s that was applied to the sensor data. The median duration of the lying bouts and the non-lying bouts that were not detected by the model was 3.0 min and 4.0 min, respectively. Median duration of lying bouts and non-lying bouts that were detected too much by the model was 6.5 min and 5.0 min, respectively.

On pasture, 44 lying bouts were classified correctly by the model and identified without interruption, both in ground truth and model output. Thus, those lying bouts could be compared regarding their duration. Median duration of the lying bouts did not differ between ground truth and model output (57.7 vs. 56.6 min) on pasture. In the barn, 37 lying bouts could be compared regarding their duration. Duration of lying bouts differed significantly (*p* < 0.01) between ground truth and model output (42.3 vs. 58.2 min).

**Figure 8:**
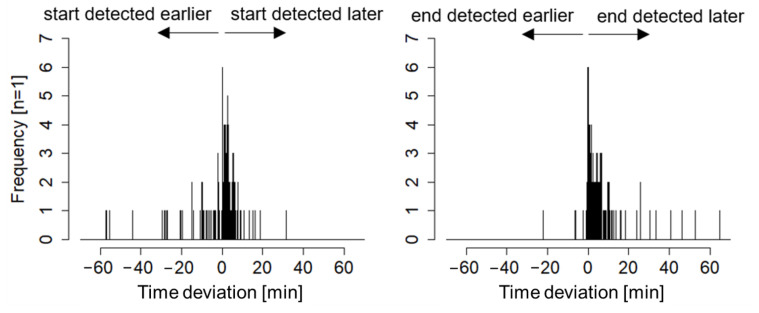
Deviation in detected beginnings (**left**) and endings (**right**) of lying bouts by the model compared to Ground Truth.

In total, the beginning of 159 and the ending of 161 lying bouts was assessed in detail. Only eight beginnings and six endings were correctly identified by the model within ±4 s compared to ground truth, but 88.6% of the beginnings and 84.4% of the endings were detected within ±10 min (see Figure 8). Most beginnings (n = 116) and endings (n = 129) were detected too late by the model with a median deviation in time of 2.3 and 4.1 min.

### 3.5. Lying Behavior in Different Husbandry Systems

For comparison of lying behavior over 24 h between husbandry systems (=farms), one day of each farm was chosen based on the availability of sensor data. The lying time of four (farm 1), nine (farm 2) and eight (farm 3) animals was included in the comparison. The mean (±standard deviation) for each farm can be observed in Table 9. The lying time on farm 3 was significantly higher than on farm 1 and farm 2 (*p* < 0.05). On farm 1 and farm 2, lying time did not differ significantly (*p* = 1.0). Although numerically higher during the night, lying time did not differ significantly (*p* = 0.20) between daytime and nighttime (4.8 vs. 7.1 h). On farm 2, lying time during the day and during the night was similar (6.2 vs. 6.8 h; *p* = 0.48). Lying time during the day was numerically but not statistically higher than during the night on farm 3 (8.2 vs. 7.8 h; *p* = 0.18).

## 4. Discussion

Behavior prediction from sensor data in order to improve welfare has been the center of previous studies. A 3D accelerometer is widely used to classify moving and non-moving behaviors such as lying and non-lying for cows [64]. Using only one sensor resulted in relatively low sensitivity and precision of collar-based systems in the studies of Martiskainen et al. [51] and Vázquez Diosdado et al. [56], but using additional sensors resolves the ambiguity between classified activities and improves prediction. Spink et al. [65] and Hanson and Mo [13] complemented accelerometer data with data from GPS sensors in order to calculate the distance travelled by cows. Based on the study from González et al. [66], GPS data can improve the accuracy of prediction; however, the frequency of data sampling plays an important role as high frequencies result in high battery consumption. In order to extract enough knowledge, in some behavior recognition systems, the magnetometer and gyroscope data are used as a complement to accelerometer data as well. Based on the related work performed by Mansbridge et al. [7] on sheep and Kamminga et al. [8] on goats where reliable prediction of animal behavior was achieved, the combination of accelerometer, magnetometer and gyroscope was chosen in our study.

In addition to the choice of sensors, defining the best sampling rate is another important step towards acquiring sensor data. High sampling rates can result in higher accuracies but reduce the battery lifespan [6]. As presented in human activity recognition studies [54,67,68,69], the goal is to reduce the sampling rate to save battery while ensuring a high classification accuracy. In order to explore sampling rate reduction in the process of model development, the sampling rate was set to 10 Hz according to previous studies [51,70]. As the further reduction in sampling rate resulted in a considerable drop in accuracy for random forest, we retained 10 Hz for the final model.

For data acquisition, we used the prototype of a monitoring system with provisional casing (see Figure 1). The casing was sufficiently robust for the temporal use within our study. For an extended application, the robustness needs to be improved. Weight and measurements—especially in relation to the animal—are notable factors in the development and validation of monitoring systems [71]. As cows with different body weights and heights were used in our study, varying systems relative to animal relations were included in the training and validation of our model. As weight and measurements will only marginally change with replacing the case, we expect no limitation to the applicability of our model afterwards.

For the training and the following evaluation of the model, ground truth data were needed. Reiter et al. [52] found a high correlation between visual and video observation for rumination behavior, proving that video observation is a reliable method for behavior data acquisition. In contrast to visual observation, more than one animal can be observed at the same time, resulting in a greater amount of data in relation to workload. In our study, the cameras on the pasture had to be moved around to capture the behavior of the moving cows without interruption, resulting in data acquisition being more laborious and requiring more operators than in the barn where the cameras were installed in fixed positions. Even though operators were present during the observation on pasture, disturbance was reduced to a minimum. Using high resolution cameras allowed the operators to keep sufficient distance from the animals. Data collection on pasture was limited to daytime as cameras without a night vision feature were used. In the barn, artificial lighting enabled acquisition of behavior data also at night. However, as there was no difference detected in performance of the model between day and night in the barn, it can be assumed that lying behavior presents equally in the sensor data independent from the time of day.

In order to generate behavior data, videos were labeled based on an ethogram, which was developed following previously published studies to ensure comparability of the results [37,51,72]. The process of labeling is time consuming, impeded by animals obscuring each other and impeded by the movement of the cameras on pasture. Detailed labeling of a subset of data allowed deeper assessment of the falsely classified time and provided data for the model development for other behavioral patterns. Using only one observer for video labeling eliminates deviations that occur between different observers. Observer reliability was satisfactory and comparable to the one obtained in the study of Ambriz-Vilchis et al. [46]. The chosen methods provided reliable and profound datasets for the development and evaluation of the model.

In the process of model development, a suitable segmentation strategy for the sensor data, i.e., an appropriate window size, must be chosen. Breaking the data into smaller window sizes can improve the classification accuracy but causes redundancy and waste of resources [6]. Walton et al. [69] investigated different window sizes in behavior prediction in sheep and found only a slight improvement of accuracy between a window size of 3 s compared to 5 s. In our study, there were only marginal differences in performance of the model between different window sizes. Due to that, 5 s were chosen as a trade-off between the accuracy and the prediction latency. A stride of 100% proved to be the most accurate for random forest. That is in line with the findings of Dehghani et al. [73], where a quantitative comparison between overlapping and non-overlapping windows showed that overlapping windows require more resources while hardly impacting the subject-independent cross-validation.

From the chosen windows, the features for the model were computed. As in our study the sensor orientation changed between farms, features independent from orientation proved to be suitable. Kamminga et al. [8] stated that sensors on animals are exposed to shifting in general and showed that models based on features independent from orientation provide high accuracy values in behavior recognition in animals.

As the last step of model development, the choice of classifier is one of the crucial parameters for animal behavior recognition. In our study, random forest scored the highest accuracy compared with decision tree, support vector machine and naive Bayes. Vázquez Diosdado et al. [56] showed that the accuracy of the decision tree is comparable with other classifiers (not including random forest), while the computational complexity is lower. Although a decision tree can perform accurately and fast on large datasets, it is prone to overfitting. Mansbridge et al. [7] compared random forest, support vector machine, k-nearest neighbor and adaptive boosting in classifying sheep behavior and showed that random forest has the highest overall accuracy. Other studies such as Rahman et al. [57] have also used the random forest classifier for behavior recognition in dairy cows. Our finding that random forest performs best is in line with the mentioned studies.

Since data resulting from the prediction model were noisy, a filter of 60 s was applied. All sequences of lying and non-lying behavior in the sensor data with shorter duration were disregarded and added to the behavior predicted before. According to González et al. [66], as lying and non-lying behavior occurs in bouts, the accuracy should be improved by considering the behavior classified before and after a sequence. In our study, only five lying bouts lasted ≤60 s and were thereby disregarded because of the filter. With representing only 1.7% of the total lying bouts, the amounts of lying bouts and the lying time were hardly affected, but the accuracy in predicting individual lying bouts improved substantially. Kok et al. [74] showed that applying a threshold of 33 s achieved the highest performance values for the detection of lying bouts while hardly affecting the measured lying time.

In general, our final model predicted lying behavior in dairy cows with a reasonable overall accuracy of 87.3%. The model was trained with data from pasture, where a higher accuracy was achieved compared to the barn (93.1 vs. 81.4%). The lower accuracy indoors is mainly based on a lower specificity. Standing (in the cubicle) was the behavior confused the most with lying by the model. Moreover, in the studies of Vázquez Diosdado et al. [56] and Martiskainen et al. [51], standing was the behavior most misclassified as lying and the other way around. The similar posture of the head and the lack of movement both while standing and lying resulted in a similar presentation of both behaviors in the sensor data and explained the difficulty of the model to distinguish precisely between the two behavioral patterns [51,75]. In addition to providing additional information on the dairy cow behavior in general [56], the training of a model for the prediction of standing up and lying down, i.e., the transition between standing and lying, could improve the distinction between lying and standing. The reduced performance in the barn can be explained by a higher standing time compared to pasture. This is supported by the negative correlation between standing time and specificity as well as accuracy per animal per day (for the days where labeling was performed in detail). Moreover, the differences in performance between the different days and the different animals can be explained with variation in standing time. In addition to heat or health disorders, dairy cows show an increased standing time when ambient temperatures are high [28]. The increase in standing time caused by heat load varies between animals. Another reason for the disparity of performance in the different cows could be a variation in movement patterns of the head and neck in between animals. The interpretation of performance values for different days and animals is limited by the fact that not all animals within one farm were observed on all observation days. On the one hand, performance per day is influenced by the performance of the model on the animals observed on that day. On the other hand, if animals were observed on days with more standing time in general, the overall performance on those animals is worse.

There are various monitoring systems available that predict different behavioral patterns in cows, including lying, with reasonable accuracy either in the barn or on pasture exclusively. Table 10 shows that our model performed well in predicting lying behavior compared to other models. However, when comparing the results, methodological differences have to be considered. In contrast to our study and the other papers, steers instead of dairy cows were used in the study of González et al. [66]. Only the system evaluated by Molfino et al. [76] achieved a higher specificity and precision than our model. Lying was merged with standing in this study, which was the behavioral pattern confused the most with lying by our model. By merging the two behaviors, the misclassification rate is reduced and the performance increases. Unlike our system, the sensors in other studies were located at the top of the neck where a counterweight is needed for the sensor to stay in its position. Additionally, the sensor is more exposed to external forces, e.g., by the feeding fence when mounted to the top of the cows’ neck. In contrast to our model, the training and the evaluation of the systems’ models were conducted on data from the same farm. Only Molfino et al. [76] evaluated a system that was developed before and thereby trained on a different farm than where the evaluation was performed. However, the training of the model is not part of the study. Evaluating the performance of a model on data from a different farm than where the data for the development were collected on proves that, in order to apply the monitoring system on a new farm, no additional training is needed. Differences in performance between animals were assessed in the study of González et al. [66]. Variation in sensitivity was wider, but smaller variation was found in precision compared to our study. Vázquez Diosdado et al. [56] did not assess deviation in performance between animals but discovered large variations in movement patterns, i.e., in the sensor data between the animals.

In all studies presented in Table 10, the models were trained in the barn and evaluated in the barn or trained on pasture and evaluated on pasture. To our knowledge, this is the first study developing and evaluating a model for a (collar-based) monitoring system for dairy cows that predicts the behavior of dairy cows reliably on pasture as well as in the barn.

Ambriz-Vilchis et al. [46] evaluated the performance of a rumination collar on cows kept in a freestall barn as well as on cows kept on pasture. Rumination times predicted by the system agreed with rumination time measured by visual and video observation on cows kept indoors. However, major differences in rumination time were found for pasture. The results are confirmed by Elischer et al. [47], who assessed the performance of a neck collar predicting feeding behavior as well as rumination behavior on cows on pasture with access to a barn. In this study, the performance was tested on the same animals inside as well as outdoors. Only moderate correlations were observed between the collar output and visual observation.

A reasonable amount of lying bouts (95.12%) was detected correctly by our model. In the study of Kok et al. [74], 99.2% of the lying bouts were detected correctly, i.e., by both the pedometers attached to one cow. In contrast to our study, lying bouts in ground truth data with a duration of less than 33 s were discarded. In the study of Ledgerwood et al. [77] a pedometer detected 99.3% of the lying bouts that were observed in ground truth without applying any filter and by using a window size of 6 s. In the presented study, a few lying bouts were not identified correctly by the model, but as the median duration of those bouts was short (3.02 min), the effect on total lying time is small. To our knowledge, there were no studies conducted on the performance of a model in detecting the beginnings and endings of individual lying bouts.

Lying time on pasture (farm 1: 12.3 h) was significantly lower than in the barn (farm 3: 15.6 h). This finding is in line with the results from Black and Krawczel [78] and Legrand et al. [79], who observed higher lying times in the barn than on pasture as well. Lower lying times on pasture are likely based on the prolonged duration of feed intake (=grazing) on pasture compared to the barn. While in the barn, dairy cows spend 3.9 to 4.2 h feeding [80,81], and grazing time occupies 10.6 to 11.1 h a day [82]. Although higher inaccuracy of our model in the barn resulted, the use of lying time predicted over 24 h in the barn has to be treated with caution. Only numerical differences were detected between lying time during the day and during the night, which is in contrast to the findings of Herbut and Angrecka [83] who observed longer lying times during the night on pasture compared to daytime. Their finding is supported by Winckler et al. [84], who had the same conclusion for lying time distribution in the barn.

## 5. Conclusions and Future Work

In conclusion, we could show that by using machine learning methods on sensor data from a motion unit, it is possible to predict lying behavior with reasonable accuracy compared to other available models and monitoring systems, especially regarding the fact that the model was applied both on data from pasture and the barn.

The models that have been trained and compared for this study were produced by state-of-the-art machine learning algorithms. The choice of these algorithms was based on related research from literature on activity recognition. We then compared the models produced by the best-performing algorithm and came to similar results that random forest models achieve the best accuracy for the given use case.

Prediction must be enhanced, particularly in the barn, in order to enable the identification of behavioral changes caused, e.g., by heat, health disorders or extreme weather conditions. To improve the performance of the model in predicting lying behavior, standing up and lying down events could be studied. By adding models for the prediction of other behavioral patterns, especially standing, the prediction of lying behavior could be improved by reducing the falsely classified data points. Additionally, more information is generated in order to relate the behavioral changes to different health challenges or estrous. The collected data can be used for the development of further models. In addition to data from the accelerometer, magnetometer and gyroscope, data from other sensors could be used. Partial GPS data have been collected from dairy cows on farm 1 where the GPS sensor functioned as the timer for the sensor data.

### Future Work

In the future, context-aware location data and GPS data could be used to improve the accuracy of our model.

The data that needs to be managed in an IoT ecosystem steadily grew in all of its three big data dimensions: volume, velocity and variety. The volume increases due to the elevating amount of data generating devices [85,86] and velocity by advances in communication technologies such as 5G [87]. Kaur et al. [88] even calls it the Internet-of-Big-data.

The processing of this huge amount of data utilizes many resources. Current IoT platforms are mainly centralized and lack the feature of resource-aware processing in the sense of edge and fog processing [89]. Centralized processing is generally suboptimal since it uses the Wide Area Network (WAN) bandwidth highly inefficiently due to sending all data to the cloud in order to process it there. Furthermore, cloud computing induces high latency, high energy consumption and privacy concerns arise. Properly positioning the processing along the process from the data sources to the sinks is the intended strategy. Enabling edge and fog processing is crucial for being resource efficient and for real-time low latency applications. Data processing in IoT is geographically distributed by the nature of the ecosystem [90,91].

## Figures and Tables

**Table 1:** Sward composition (grasses, herbs and legumes) of pastures on farm 1 and farm 2.

	Farm 1	Farm 2
Grasses (%)	64	30
- *Lolium perenne*	47	24
- *Poa pratensis*	9	5
- *Festuca pratensis*	8	1
- *Poa trivialis*	<1	<1
Legumes (%)	36	70
- *Trifolium repens*	36	70
Herbs (%)	3	2
- *Plantago major*	2	1
- *Taraxacum* sect. *Ruderalis*	<1	<1
- *Bellis perennis*	<1	<1

**Table 2:** Ethogram of dairy cow behavior used for labeling the video data in order to generate ground truth data. Definitions were derived from Martiskainen et al. [51], Reiter et al. [52] and Werner et al. [53].

Behavior	Definition
Lying	The body of the animal is not supported by any limb. The sternum and/or the belly are/is in contact with the ground. The limbs are bent or stretched out.
Lying down	The transition from standing/walking to lying. From bending one forelimb to completely lying.
Standing up	The transition from lying to standing/walking. From stretching the shoulders to standing on four limbs/walking.
Lying bout	Time between a lying down and a standing up event.
Standing - In the cubicle; - In the alley; - At the feeding table; - In the feeder.	The body of the animal is supported by at least three limbs: - At least two feet are located in the cubicle; - At least three feet are located in the alley; - The head is above the feeding table; - All four feet are located within the area of the feeder.
Walking	The animal moves forward or backwards at walking pace and makes two or more consecutive steps in one direction.
Grazing	The animal bites off grass, chews and swallows it and moves forward with a lowered head. From the first grip of grass to the lifting of the head higher than the carpal joint.
Feeding	The muzzle of the animal is located beneath the lower margin of the feeding fence and in the feed.
Chewing	The animal moves its lower jaw in a grinding movement without having regurgitated before.
Ruminating	The animal regurgitates food bolus, chews and swallows it. From regurgitating the first bolus to swallowing the last bolus.
Drinking	The muzzle of the animal is located below the outer margin of the trough consuming water.
Other	Social, comfort, exploration and fly repellent behavior.
Idle time	The animal is not visible in the video image or covered by another animal.

**Table 3:** Classifiers with corresponding selected hyperparameters. The hyperparameters were tuned by an exhaustive grid search method in order to find the optimized parameters for each model.

Classifier	Hyperparameters
Random Forest	Number of trees: 95 Criterion: Gini (calculates the probability of a specific feature classified incorrectly when selected randomly) Splitter: choose the best split at each node Maximum depth of tree: 25 Minimum number of samples required to split an internal node: 2 Minimum number of samples required to be a leaf node: 1 Maximum number of features used at each split: 6
Decision Tree	Criterion: Gini Maximum depth of the tree: 5 Minimum number of samples required to split an internal node: 2 Minimum number of samples required to be a leaf node: 1
Support Vector Machine	Kernel: Radial Basis Function—RBF Regularisation parameter: 10 Kernel Coefficient (gamma): Scale
Naive Bayes	Type: Gaussian

**Table 4:** Behavior instances distribution used for training the model. The training dataset contains the data of three animals from farm 1.

	Amount of Instances	Instances (%)
Grazing	778,867	51.0
Lying	491,522	32.2
Walking	116,175	7.6
Standing	139,707	9.2
Total	1,526,271	100.0

**Table 5:** Amount of datasets collected for the evaluation of the model. Differences in amount of collected datasets are based on different availability and runtime of sensors between farms, rounds and days.

	Farm	1	2		3
	Location	Pasture	Pasture	Barn	Barn
	Total (h)	106.0	132.7	51.4	186.2
Round 1	Day 1 (h) (no. of cows)	14.4 (3)	32.2 (11)	23.3 (10)	20.2 (4)
	Day 2 (h) (no. of cows)	27.5 (5)	44.8 (11)	25.9 (10)	41.2 (7)
	Day 3 (h) (no. of cows)	-	55.7 (10)	2.2 (3)	65.7 (8)
	Day 4 (h) (no. of cows)	-	-	-	59.1 (7)
Round 2	Day 1 (h) (no. of cows)	36.1 (6)	-	-	-
	Day 2 (h) (no. of cows)	28.0 (6)	-	-	-

**Table 6:** The two selected models based on the random forest classifier with two different sets of features (orientation-dependent/orientation-independent).

	Model 1	Model 2
Classifier	Random Forest	Random Forest
Window size	5 s	5 s
Feature set	24 features	36 features
	This model is predicted to be sensor orientation sensitive	This model is known to be insensitive to sensor orientation

**Table 7:** Accuracy of the two models with different feature sets (orientation-dependent/orientation-independent) when both trained and tested on farm 1 (=same sensor orientation).

		Model 1 (%)	Model 2 (%)
Train-valid accuracy	Mean	95.8	92.2
	SD	0.5	0.8
Test accuracy	Mean	88.7	92.0
	SD	5.6	2.8

**Table 8:** Accuracy of the two models with different feature sets (orientation-dependent/orientation-indipendent) when trained on farm 1 and tested on farm 2 (=different sensor orientation).

		Model 1 (%)	Model 2 (%)
Train-cross accuracy	Mean	95.7	92.1
	SD	0.5	0.7
Test accuracy	Mean	73.9	91.1
	SD	20.8	4.3

**Table 9:** Comparison of lying times (mean ± standard deviation) over 24 h and during daytime (06.00 to 18.00 h) and nighttime (18.00 to 06.00 h) between the different farms (=husbandry systems).

	Farm 1	Farm 2	Farm 3
lying time/d (h)	12.3 (±0.8)	12.2 (±0.6)	15.6 (±1.3)
lying time daytime (h)	4.8 (±0.6)	6.2 (±0.5)	8.2 (±0.6)
lying time nighttime (h)	7.1 (±1.9)	6.8 (±1.3)	7.8 (±1.0)

**Table 10:** Performance of the model compared to the results of other model evaluations. ${}^{a}$ Martiskainen et al. [51]. ${}^{b}$ Vázquez Diosdado et al. [56]. ${}^{c}$ Molfino et al. [76]. ${}^{d}$ González et al. [66]. ^e^ Lying behavior includes ruminating while lying, but excludes standing. ${}^{f}$ Lying behavior excludes ruminating and standing. ${}^{g}$ Lying behavior includes standing, but excludes ruminating. ${}^{h}$ Training not included in the paper (=conducted on different farm). ${}^{i}$ Variation due to different classifiers and different window sizes.

	Our Model		1 ${}^{a}$	2 ${}^{b}$	3 ${}^{c}$	4 ${}^{d}$
Husbandry system	Pasture	Barn	Barn	Barn	Pasture	Pasture
Definition lying behavior	1 ^e^		2 ${}^{f}$	1 ^e^	3 ${}^{g}$	3 ${}^{g}$
Sensor(s)	Accelerometer + Magnetometer + Gyroscope		Accelerometer	Accelerometer	Accelerometer	Accelerometer + GPS
Sensor position	Lower neck		Top of the neck	Top of the neck	Top of the neck	Top of the neck
Training data	One farm		One farm	One farm	- ${}^{h}$	One farm
Evaluation data	Same farm + two others		Same farm	Same farm	One farm	Same farm
Sensitivity	95.6%		80%	55.4–92.9% ${}^{i}$	77%	86.3%
Specificity	80.1%		-	-	99%	94.8%
Precision	80.5%		83%	85.4–96.6% ${}^{i}$	93%	-
Accuracy	93.1%	81.4%	84 %	-	-	92.5%

## Data Availability

Data is contained within the article.

## Abbreviations

The following abbreviations are used in this manuscript:

PLF	Precision Livestock Farming;
IoT	Internet of Things;
DIM	Days in milk;
BCS	Body condition score;
3D	three-dimensional;
GPS	General Positioning System;
RTC	Real-time clock;
TP	True positive;
TN	True negative;
FP	False positive;
FN	False negative.
